# A novel proteasome inhibitor suppresses tumor growth *via* targeting both 19S proteasome deubiquitinases and 20S proteolytic peptidases

**DOI:** 10.1038/srep05240

**Published:** 2014-06-10

**Authors:** Ningning Liu, Chunjiao Liu, Xiaofen Li, Siyan Liao, Wenbin Song, Changshan Yang, Chong Zhao, Hongbiao Huang, Lixia Guan, Peiquan Zhang, Shouting Liu, Xianliang Hua, Xin Chen, Ping Zhou, Xiaoying Lan, Songgang Yi, Shunqing Wang, Xuejun Wang, Q. Ping Dou, Jinbao Liu

**Affiliations:** 1 State Key Lab of Respiratory Disease, Protein Modification and Degradation Lab, Department of Pathophysiology, Guangzhou Medical University, Guangdong 510182, China; 2 Guangzhou Research Institute of Cardiovascular Disease, The Second Affiliated Hospital of Guangzhou Medical University, Guangzhou, Guangdong 510260, People's Republic of China; 3 Division of Basic Biomedical Sciences, Sanford School of Medicine of the University of South Dakota, Vermillion, South Dakota 57069, USA; 4 The Molecular Therapeutics Program, Barbara Ann Karmanos Cancer Institute, and Departments of Oncology, Pharmacology and Pathology, School of Medicine, Wayne State University, Detroit, Michigan 48201-2013, USA; 5 These authors contributed equally to this work.

## Abstract

The successful development of bortezomib-based therapy for treatment of multiple myeloma has established proteasome inhibition as an effective therapeutic strategy, and both 20S proteasome peptidases and 19S deubiquitinases (DUBs) are becoming attractive targets of cancer therapy. It has been reported that metal complexes, such as copper complexes, inhibit tumor proteasome. However, the involved mechanism of action has not been fully characterized. Here we report that (i) copper pyrithione (CuPT), an alternative to tributyltin for antifouling paint biocides, inhibits the ubiquitin-proteasome system (UPS) *via* targeting both 19S proteasome-specific DUBs and 20S proteolytic peptidases with a mechanism distinct from that of the FDA-approved proteasome inhibitor bortezomib; (ii) CuPT potently inhibits proteasome-specific UCHL5 and USP14 activities; (iii) CuPT inhibits tumor growth *in vivo* and induces cytotoxicity *in vitro* and *ex vivo*. This study uncovers a novel class of dual inhibitors of DUBs and proteasome and suggests a potential clinical strategy for cancer therapy.

The ubiquitin-proteasome system (UPS) is a tightly regulated process responsible for maintenance of protein homeostasis in cells. The UPS has proven to be an important target for cancer treatment. Indeed, targeting this pathway has been validated as a strategy by Food and Drug Administration (FDA) approval of bortezomib for the treatment of multiple myeloma and mantle cell lymphoma in 2003^1^. Many patients have benefited from bortezomib-based therapy, and the overall survival rate of multiple myeloma has been significantly increased in the last decade. However, there are several shortcomings associated with the use of bortezomib, including relapses in the treated patients, severe toxicities, and little or no clinical activity against solid tumors^2^,^3^. Therefore, there is a need to discover new proteasome inhibitors.

A metal compound, cisplatin, has contributed to the cure or treatment of many cancers since its inception, but toxicities and resistance associated with cisplatin treatment have hindered its clinical use and spurred the search for new, less toxic metal-based treatments^4^,^5^. Biologically active metals like copper, as well as other metals like gold have been used successfully in the treatment of other ailments, with different mechanisms of action, such as inhibition of the UPS^6^,^7^,^8^. Several copper-chelating compounds have been investigated for their ability to bind copper and inhibit the cellular proteasome to induce tumor cell apoptosis^9^,^10^,^11^,^12^. We and others have examined the possible chemotherapeutic properties of diethyldithiocarbamate (EtDTC) complexes^11^,^13^. When EtDTC were coupled with copper and zinc, we found that both Zn (EtDTC)_2_ and Cu (EtDTC)_2_ complexes exhibited inhibitory activity against purified 20S proteasome and intact 26S proteasome in cancer cells. Zinc- and copper-(EtDTC)_2_ complexes were found to be more active against 26S proteasome than 20S proteasome^7^,^11^. Therefore, it has been hypothesized that copper or zinc complex inhibits the proteasome by a mechanism that is distinct to the clinically used drug bortezomib, targeting the 19S rather than the 20S proteasome^7^,^11^, but this hypothesis has not been tested. In humans, three deubiquitinases (DUBs) are associated with the 19S regulatory particles. Two of these, UCHL5/Uch37 and USP14/Ubp6, are cysteine proteases and belong to the ubiquitin C-terminal hydrolases (UCH) and ubiquitin specific proteases (USP) families, respectively. The third is POH1 (RPN11), a Zn^2+^-dependent protease of the JAB1/MPN/Mov34 (JAMM) family localized within the lid of the 19S proteasome^14^. The physiological roles of the 19S DUBs are not completely clear even though some studies have reported the important roles of proteasome DUBs in protein degradation^15^,^16^,^17^,^18^.

Pyrithione (PT) possesses excellent metal chelating properties^19^,^20^,^21^. In this current work, the effects of copper, PT and their complex, copper pyrithione (CuPT), on UPS function and cell viability were investigated. We provide direct evidence that CuPT inhibits the UPS *via* inhibiting both proteasome-specific DUBs and 20S proteasome peptidases and this inhibition plays an important role in CuPT-mediated cytotoxicity, unveiling a novel mechanism for the anti-cancer effects of metal-containing compounds.

## Results

### PT and CuCl_2_ in combination synergistically enhanced cytotoxicity

**Figure 1:**
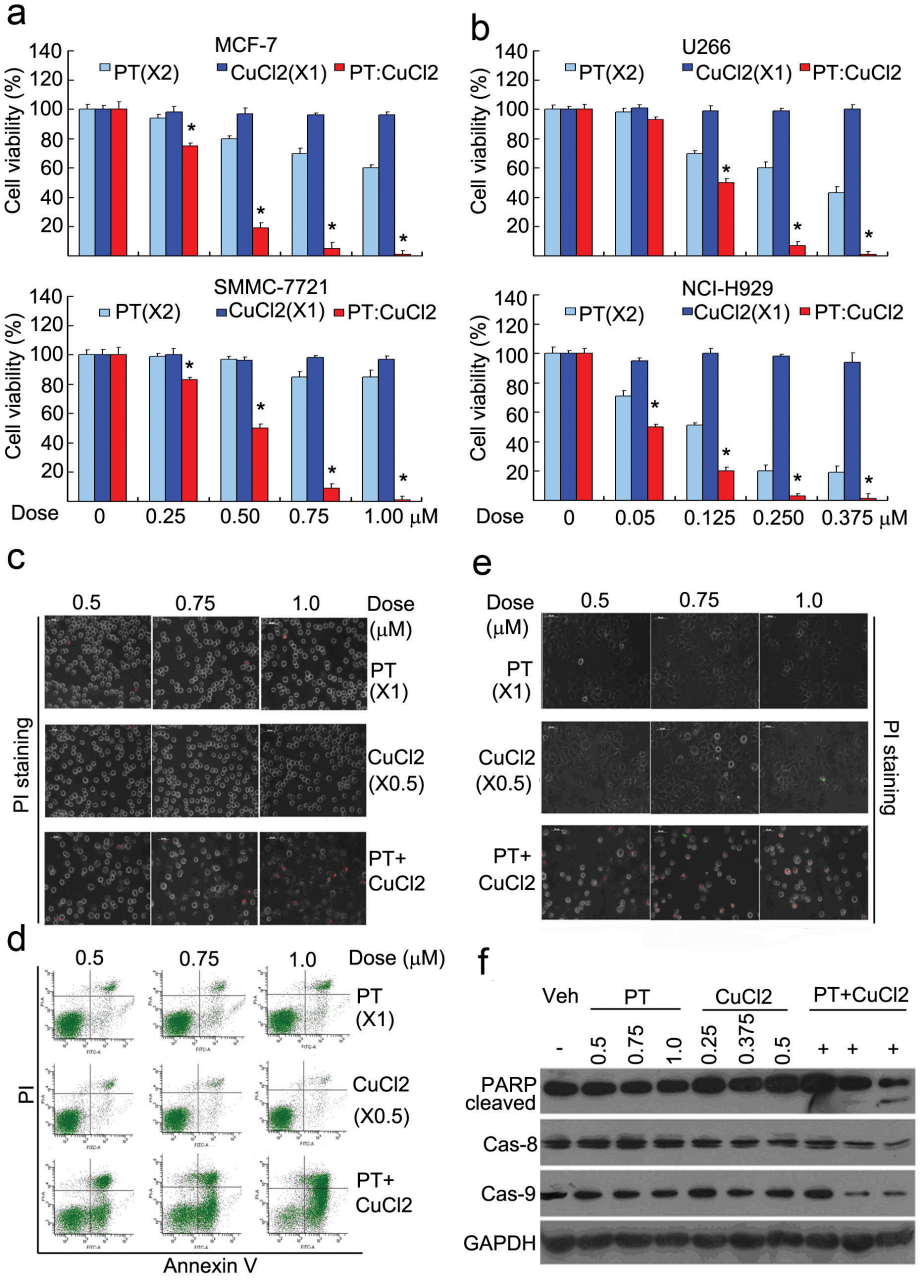
Pyrithione (PT) and CuCl2 in combination enhanced cytotoxicity. (a and b) PT and CuCl_2_ synergistically decreased cell viability. Cancer cells (MCF-7, HepG2, U266, NCI-H929) were treated with PT, CuCl_2_ alone and their combination (PT/CuCl_2_: 2:1) at the indicated doses for 24 hours, cell viability was detected by MTS assay. Mean ± SD (n = 3). *P < 0.05, *versus* each treatment alone. (c and d) PT and CuCl_2_ in combination accelerated cell apoptosis and cell death in U266 cells. U266 cells were exposed to PT, CuCl_2_ and their combination at the indicated doses for 24 hours, cell death and cell apoptosis were detected by either PI staining with an inverted fluorescence microscope in live cells (c) or by Annexin V/propidium (PI) staining with flow cytometer (d). Scale bar = 50 μm. (e and f) PT and CuCl_2_ in combination accelerated cell death, PARP cleavage and caspase activation in MCF-7 cells. MCF-7 cells were incubated with various doses of PT, CuCl_2_ and their combination, then cell death was detected with PI staining in live cells (24 hours), and caspase-8, -9, PARP cleavage were detected by Western blot (12 hours). GAPDH: loading control. Scale bar = 50 μm.

We first investigated the cytotoxic effects of PT plus copper on cancer cells. At 24 hours after treatment, cell viability detected by the MTS assay was not discernibly affected by CuCl_2_ alone, modestly decreased by PT alone, but dramatically decreased by 2:1 PT/CuCl_2_ combination treatment with IC_50_ values of 0.175, 0.125, 0.25, and 0.05 μM in MCF-7, HepG2, U266 and NCI-H929 cancer cell lines, respectively (Figures 1a and b). Also, compared to PT or CuCl_2_ alone, the PT/CuCl_2_ combination treatment induced cell death more effectively. This is evidenced, for example, by the result from 24 hour treatment of U266 cancer cells, followed by live cell propidium iodide (PI) staining (Figure 1c) and by Annexin V/PI staining followed by flow cytometry (Figure 1d). Similarly, PT/CuCl_2_ treatment for 24 hours induced high levels of PI-positivity in MCF-7 breast cancer cells, compared to PT or copper alone (Figure 1e) and such a treatment for 12 hours also induced PARP cleavage and decreases of full-length caspase 8 and caspase 9 (Figure 1f). These results demonstrate that the combination of PT and CuCl_2_ induces cytotoxicity in multiple cancer cell lines much more effectively than PT or CuCl_2_ alone.

### PT and H_2_O_2_ in combination synergistically enhanced cytotoxicity

**Figure 2:**
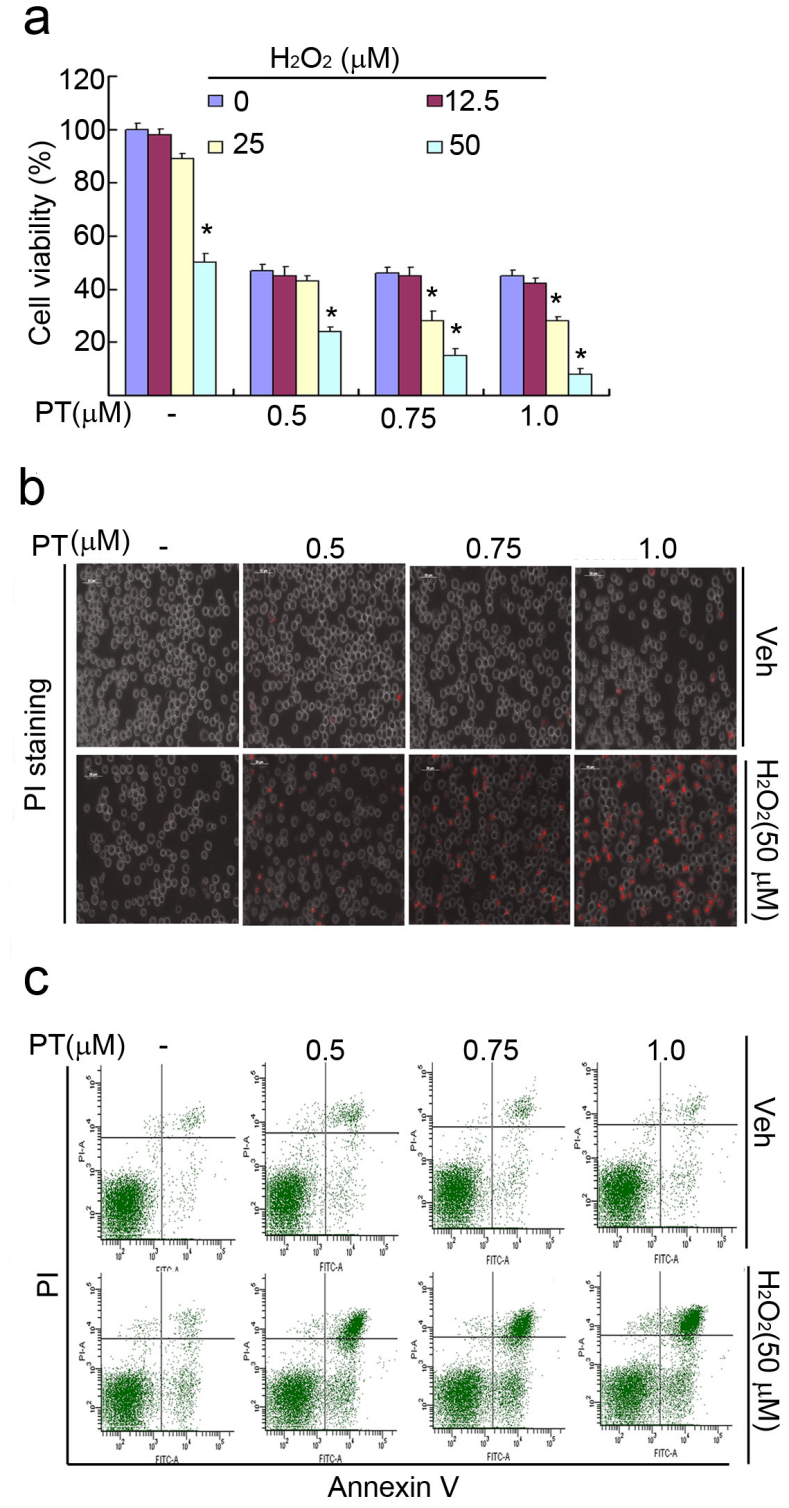
Pyrithione (PT) and H_2_O_2_ in combination enhanced cytotoxicity. (a) PT and H_2_O_2_ synergistically decreased cell viability. U266 cancer cells were treated with PT, H_2_O_2_ alone and their combination at the indicated doses for 24 hours, cell viability was detected by MTS assay. Mean ± SD (n = 3). *P < 0.05, *versus* each PT treatment alone. (b and c) PT and H_2_O_2_ in combination accelerated cell apoptosis and cell death in U266 cells. U266 cells were exposed to PT, H_2_O_2_ and their combination at the indicated doses for 24 hours, cell death and cell apoptosis were detected by either PI staining with an inverted fluorescence microscope in live cells (b) or by Annexin V/propidium (PI) staining with flow cytometer (c). Scale bar = 50 μm.

Since CuCl_2_ is a strong oxidant, here we used another oxidant H_2_O_2_ instead of CuCl_2_ in combination with PT to investigate their cytotoxic effect in cancer cells. U266 cancer cells were treated with PT, H_2_O_2_ alone and their combination at the indicated doses for 24 hours. The enhanced decrease of cell viability was observed with the treatment of PT combining with H_2_O_2_ at the doses of 25 and 50 μM but not at the low dose of 12.5 μM (Figure 2a); cell death was dramatically accerelated with the combination treatment of PT and H_2_O_2_ (50 μM) as detected by recording the PI-positive cells under a fluorescence microscope (Figure 2b) or by flow cytometry with Annexin V/PI staining (Figure 2c). These results clearly show that PT and H_2_O_2_ in combination enhanced cytotoxicity. However, whether PT + H_2_O_2_ uses the identical mechanism of action as that of PT + CuCl_2_ needs to be further investigated. Indeed, we found that PT + CuCl_2_, but not PT + H_2_O_2_ induced inhibition of the UPS (see below).

### CuPT, the chelating product of PT and CuCl_2_, induced cytotoxicity in multiple cancer cells and in primary monocytes from patients with acute myeloid leukemia (AML)

**Figure 3:**
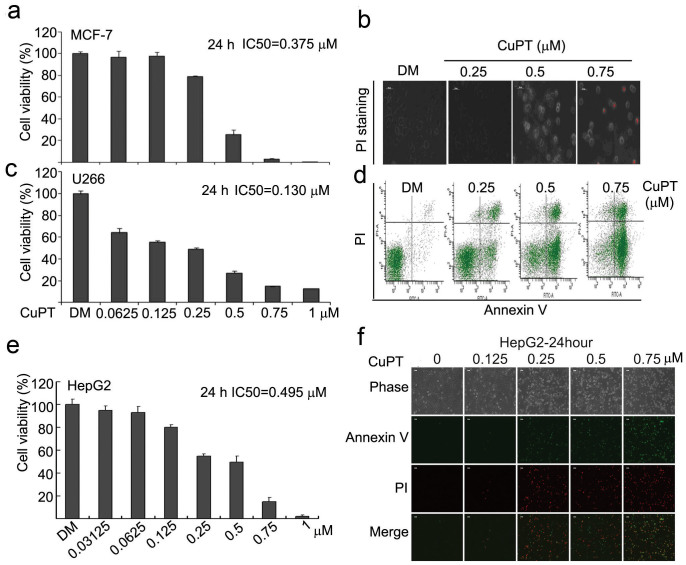
Copper pyrithione (CuPT), the chelating product of PT and CuCl2 induced cytotoxicity in multiple cancer cells. (a and b) CuPT decreased cell viability and induced cell death in MCF-7 cells. MCF-7 cells were treated with CuPT for 24 hours, and then cell viability and cell death were detected as described above. IC_50_ was calculated and shown in (a) and representative PI-positive morphological images were shown in (b). Scale bar = 50 μm. (c and d) CuPT decreased cell viability and induced cell apoptosis in U266 cells. U266 cells were treated with CuPT for 24 hours, and then cell viability and cell apoptosis were detected. IC_50_ was calculated and shown in (c), and representative cell apoptosis flow images were shown in (d). (e and f) CuPT decreased cell viability and induced cell apoptosis in HepG2 cells. HepG2 cells were treated with CuPT for 24 hours, and then cell viability and cell apoptosis were detected. IC_50_ was calculated and shown in (e), and representative Annexin V/PI-positive and morphological images were shown in (f). Scale bar = 50 μm.

CuPT is formed from the combining of one copper with 2 PTs. We then further investigated the effect of CuPT, the complex of PT and CuCl_2_, on cytotoxicity in cancer cell lines. We found, CuPT treatment for 24 hours decreased cell viability (IC_50_: 0.375 μM, Figure 3a) and induced cell death (Figure 3b) in MCF-7 cells; in U266 cells, CuPT treatment for 24 hours decreased cell viability with an 0.130 μM of IC_50_ value (Figure 3c) and induced apoptosis as detected with Annexin V/PI staining by flow cytometry (Figure 3d); similarly, CuPT treatment also decreased cell viability (IC_50_: 0.495 μM, Figure 3e) and induced apoptosis (Figure 3f) in HepG2 cancer cells.

**Figure 4:**
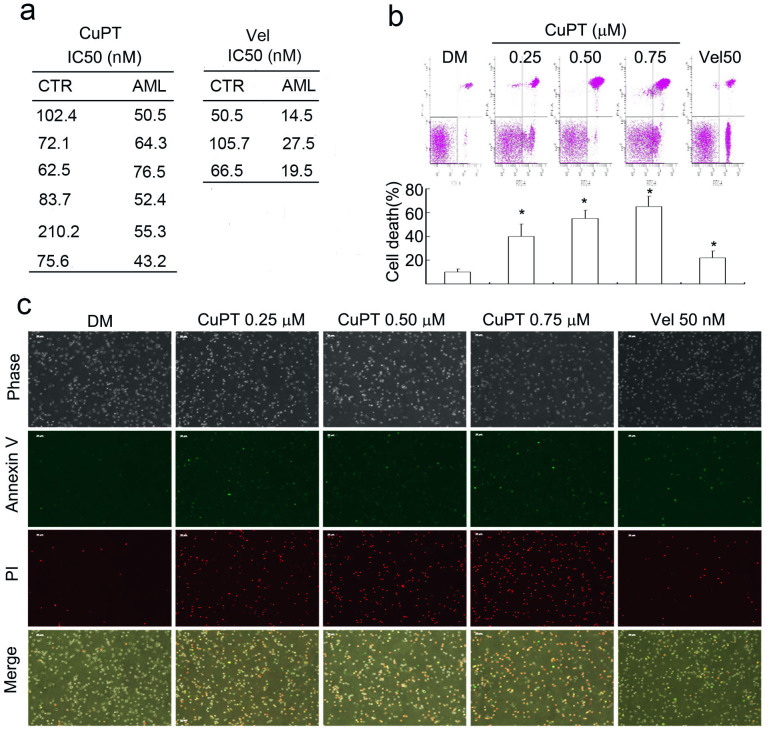
CuPT induces cytotoxicity in primary cancer cells from patients with acute myeloid leukemia (AML). (a) Cancer cells from 6 AML patients and peripheral blood mononuclear cells from 6 healthy volunteers were treated with CuPT (0.0625, 0.125, 0.25, 0.5 μM) or bortezomib (Vel, 50 nM) for 24 hours and the cell viability was detected by MTS assay. Mean ± SD (n = 3). *P < 0.05, *versus* DMSO control. (b and c) Cancer cells from 3 AML patients were isolated and incubated with CuPT (0.25, 0.5, 0.75 μM) or Vel (50 nM) for 12 hours. Cell apoptosis was analyzed by flow cytometry and by imaging under a fluorescence microscope. Representative flow images were shown in and the cell death data summarized (b). Mean ± SD (n = 3). *P < 0.05, *versus* DMSO control. The phase contrast and fluorescent images were taken and merged. Typical images were shown (c). Red indicates PI-positive and green indicates Annexin V-positive. Scale bar = 50 μm.

The *ex vivo* cytotoxic effects of CuPT on bone marrow cells obtained from patients with AML and peripheral blood mononuclear cells from healthy volunteers (CTR) were also evaluated. CuPT and bortezomib decreased cell viability of primary monocytes from AML patients (AML) with an average IC_50_ value 57.03 and 20.50 nM, respectively, while in normal controls (CTR) the average IC_50_ values were estimated at 101.08 and 74.23 nM, respectively (Figure 4a). CuPT treatment for 12 hours at doses ranging from 0.25 to 0.75 μM resulted in significant apoptosis in the monocytes from AML patients as detected with Annexin V/PI staining by flow cytometry (Figure 4b) or by fluorescence microscope (Figure 4c). These results demonstrate the *in vitro* inhibitory effect of CuPT on cancer cells.

### PT plus CuCl_2_ but not H_2_O_2_ synergistically induces the inhibition of the UPS

**Figure 5:**
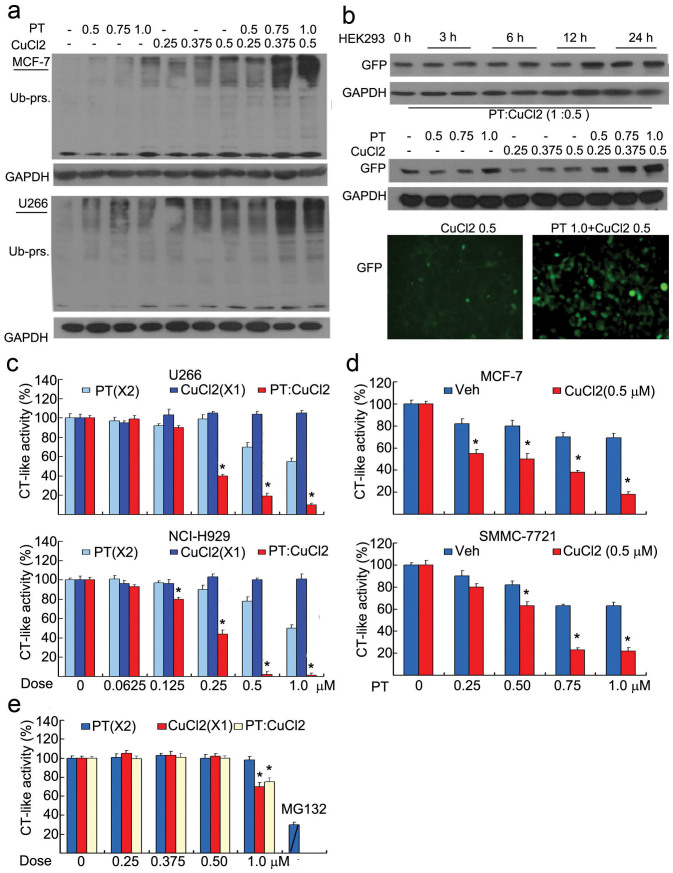
PT plus CuCl2 but not H_2_O_2_ enhanced the inhibition of the ubiquitin-proteasome system. (a) PT plus CuCl_2_ enhanced the accumulation of ubiquitinated proteins. MCF-7 and U266 cells were treated with various doses of PT, CuCl_2_ and their combination for 12 hours, and then ubiquitinated proteins were detected by Western blot. GAPDH: loading control. (b) PT plus CuCl_2_ induced GFPu accumulation. HEK-293 cells stably harboring GFPu, a surrogate proteasome substrate, were treated with PT, CuCl_2_ and their combination for 12 hours, and then GFPu was detected either by Western blot or by inverted fluorescence microscope. (c) U266 and NCI-H929 cancer cells were exposed to PT, CuCl_2_ and the combination (PT/CuCl_2_: 2:1) for 6 hours, and then CT-like substrate was added to the treated cells and the CT-like activity was measured by a multiple plate reader. Mean ± SD (n = 3). *P < 0.05, *versus* each treatment alone. (d) MCF-7 and SMMC-7721 cancer cells were treated with increasing doses of PT in the absence or presence of 0.5 μM CuCl_2_ for 6 hours, CT-like activity was measured as in (c). Mean ± SD (n = 3). *P < 0.05, *versus* PT treatment alone. (e) 20S proteasome was incubated with PT, CuCl_2_ and their combination (2:1), and then CT-like activity was measured. MG132 (3 μM) was used as a positive control. Mean ± SD (n = 3). *P < 0.05, *versus* PT treatment alone. (f) PT and H_2_O_2_ in combination did not induce the accumulation of ubiquitinated proteins. U266 cells were incubated with various doses of PT, H_2_O_2_ (50 μM) and their combination for 12 hours, then ubiquitinated proteins were detected by Western blot. GAPDH: loading control.

We next investigated the effect of the CuPT on the UPS. In both MCF-7 and U266 cells, the combination treatment with PT and CuCl_2_ (2:1) for 12 hours induced high levels of ubiquitinated proteins compared with PT and CuCl_2_ treatment alone (Figure 5a). In GFPu-HEK-293 cells stably harboring GFPu, a surrogate proteasome substrate, the CuPT treatment for 12 hours accumulated high levels of GFPu protein as detected by either Western blot (Figure 5b, upper) or by fluorescence microscope (Figure 5b, lower). We next detected the effects of PT plus CuCl_2_ on proteasome CT-like peptidase activity *in situ*. It was found that in U266 and NCI-H929 cancer cells, PT alone could moderately and the combination of PT/CuCl_2_ (2:1) dramatically inhibited the CT-like activity, while CuCl_2_ alone could not inhibit the CT-like peptidase activity (Figure 5c); in MCF-7 and SMMC-7721 cancer cells, PT could cause a dose-dependent inhibition of CT-like activity in the presence of 0.5 μM CuCl_2_ (Figure 5d). The direct effect of PT plus CuCl_2_ on a purified 20S proteasome was further measured *in vitro*. PT, CuCl_2_ (less than 0.5 μM) and their combination did not have any inhibitory effect on CT-like activity *in vitro* (Figure 5e). 1 μM of CuCl_2_ and its combination with PT could inhibit CT-like activity to some extent. Contrary to combining with CuCl_2_, the combination of PT and H_2_O_2_ did not induce the accumulation of ubiquitinated proteins (Figure 5f).

### CuPT induces UPS malfunction

**Figure 6:**
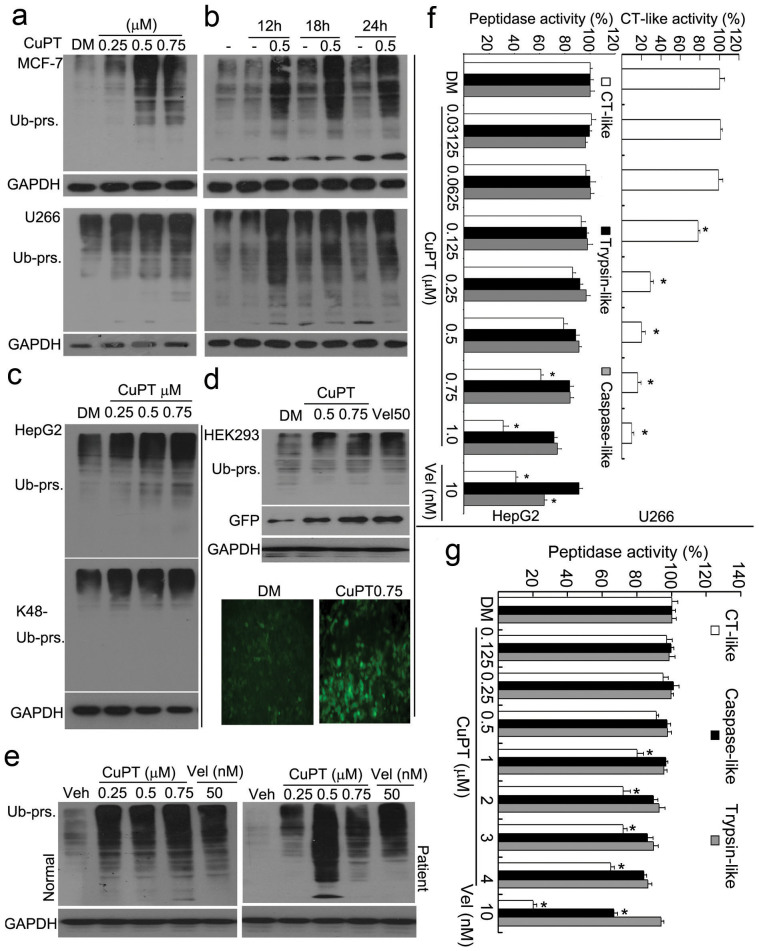
Therapeutic doses of CuPT inhibits the proteasome function in cultured cells. (a and b) CuPT induced the accumulation of ubiquitinated proteins. MCF-7 and U266 cells were treated either with CuPT (0.5, 0.75, 1.0 μM) for 12 hours or CuPT (0.5 μM) for various times (12, 18, 24 hours), and then ubiquitinated proteins were detected by Western blot. Dose- or time-dependent effect of CuPT was shown in (a) and (b), respectively. (c) CuPT induced the accumulation of K48-linked poly-ubiquitinated proteins. HepG2 cells were exposed to CuPT as indicated for 12 h, ubiquitinated and K48-linked proteins were detected by Western blot. (d) CuPT induced the accumulation of GFPu, a surrogate proteasome substrate. HEK-293 cells harboring GFPu were treated with CuPT for 12 hours, and then ubiquitinated proteins and GFP protein were detected by Western blot or imaged under an inverted fluorescence microscope. Bortezomib/Velcade (Vel) was used a positive control. (e) CuPT induced the accumulation of ubiquitinated proteins in primary cultured cancer cells. Cancer cells from AML patients or normal controls were cultured and treated with various doses of CuPT and Vel for 6 hours, and then the ubiquitinated proteins were detected by Western blot. (f) The effect of CuPT on proteasome peptidase activity in situ. HepG2 cells or U266 cells were treated with increasing doses of CuPT and Vel (10 nM) for 6 hours, followed by addition of proteasome substrates to the treated cells, and then peptidase activities including CT-like, caspase-like and trypsin-like were detected *in situ*. Mean ± SD (n = 3). *P < 0.05, *versus* control-treated. (g) The effect of CuPT on proteasome peptidase activities *in vitro*. 20S proteasome was treated with increasing doses of CuPT or Vel *in vitro* and then proteasome peptidase activities were detected.

We next tested whether UPS inhibition induced by the combination of PT and copper relied on the formed product, CuPT. To do so, CuPT was synthesized in our laboratory and used for the below experiments. Endogenous proteasome substrate proteins were first detected in human U266 and MCF-7 cancer cells to assess the effect of the synthetic CuPT on the UPS. We found that 12 h CuPT treatment at 0.5, 0.75 and 1.0 μM and 0.5 μM CuPT treatment for 12, 18 and 24 hours dramatically induced the accumulation of ubiquitinated proteins in MCF-7 and U266 cell lines (Figure 6a and b). In HepG2 cells, CuPT induced the accumulation of both total and K48-linked ubiquitinated proteins (Figure 6c). Additionally, CuPT markedly increased the accumulation of a surrogate proteasome substrate, GFPu, and ubiquitinated proteins in a stable GFPu-HEK293 cell line (Figure 6d). In primary cells from either AML patients or normal controls, CuPT induced the accumulation of ubiquitinated proteins, which are comparable to 50 nM bortezomib treatment (Vel; Figure 6e). These results support that synthetic CuPT, similar to the mixture of PT and copper, could inhibit the UPS. Further results found that CuPT could dose-dependently inhibit CT-like activity but not caspase-like and trypsin-like activities, as detected by an *in situ* peptidase assay in live HepG2 and U266 cells (Figure 6f), and that CuPT only at high doses (>1 μM) inhibited the CT-like but not caspase-like and trypsin-like activities of purified 20S proteasome under cell-free condition (Figure 6g).

### CuPT inhibits proteasome deubiquitinase function

**Figure 7:**
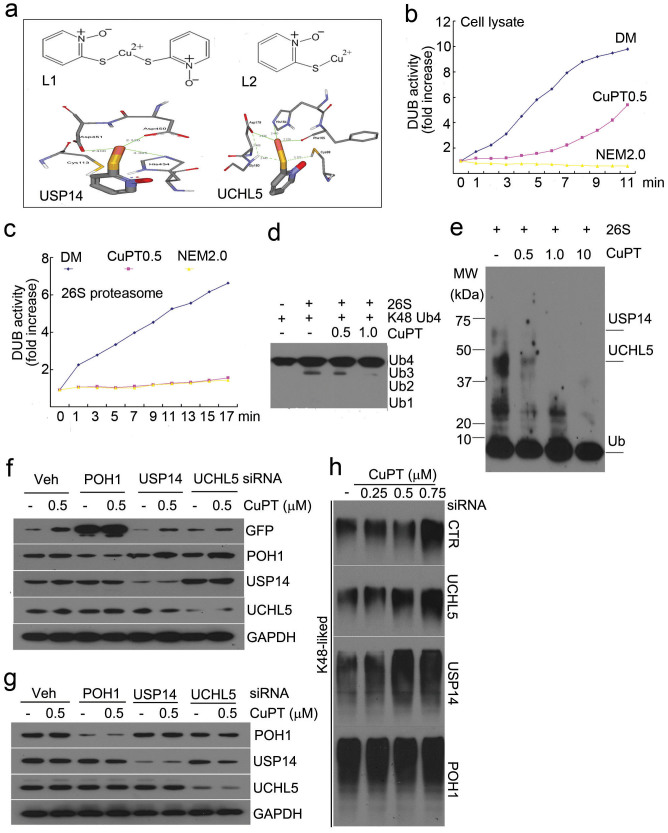
CuPT inhibits the proteasome deubiquitinase function. (a) Computational molecular docking of Cu^2+^ with POH1, UCHL5 and USP14 of 19S proteasomes. The following data were shown: the structure of copper pyrithione (L1); the structure of copper pyrithione intermediate (L2); the binding modes of compound L2 at the active site of USP14; the binding models of compound L2 at the active site of UCHL5. (b) HepG2 cell lysates were treated with CuPT (0.5 μM) and N-ethylmaleimide (NEM, 2 mM) and DUB activity was measured. Each point represents the average of 3 wells. (c) Therapeutic dose of CuPT directly inhibits 26S deubiquitinase activity. 26S proteasome was incubated with CuPT (0.5 μM), and then DUB activity was measured. Time-dependent activities were shown. Each point represents the average of 3 wells. (d) CuPT inhibits the ubiquitin chain disassembly. K48-linked ubiquitin tetramers was incubated with 26S proteasomes in the absence or presence of CuPT (0.5, 1.0 μM) for 30 min, and then ubiquitins were detected by Western blot. (e) Active-site-directed labeling of proteasomal deubiquitinases. 26S proteasomes were treated with CuPT (0.5, 1.0, 10 μM) and then labeled with HA-UbVS. Labeled HA was detected by Western blot. (f) Knockdown of proteasome DUBs affected the efficiency of CuPT on GFPu protein degradation. HEK-293 cells harboring GFPu protein were transfected with siRNA of POH1, UCHL5 and USP14 for 48 hours, and then were exposed to 0.5 μM of CuPT for 9 hours. POH1, UCHL5, USP14 and GFPu were detected by Western blot. (g and h) The effect of proteasome DUB knockdown on the accumulation of K48-linked ubiquitinated proteins. HepG2 cells were transfected with DUB siRNA for 48 hours, followed by CuPT treatment for 6 hours. DUB proteins (g) and K48-linked ubiquitinated proteins (h) were detected by Western blot.

To determine whether CuPT could target and inhibit 19S DUBs, we first performed a computational study to predict the docking affinity between CuPT and the 19S DUBs. Previous studies revealed that the catalytic core in the active site of USP14 is formed by Cys113, His434 and Asp450^22^, and that of UCHL5 is formed by Cys88, His164 and Asp179^23^. The chemical structures of CuPT (L_1_) and its mediated product (L_2_) were shown in Figure 7a. These docking results suggest that compound L_2_ could bind to the active sites of the cysteine-containing DUBs (USP14, UCHL5), with CDOCKER Interaction Energy of −40.63 and −39.11 kcal/mol, respectively. Accordingly, the binding modes of compound L2 at the active sites of the two DUBs were displayed in Figure 7a. It is noted that, in active sites, the Cu^2+^ of compound L_2_ can form networks of coordination bonds with the DUBs. In the binding site of USP14, the side chains of His434, Asp450 and Asp451 coordinate to Cu^2+^ with distances of 3.385 Å, 2.320 Å and 2.400Å, respectively. Similarly, in the binding site of UCHL5, there are three coordination bonds between the Cu^2+^ and three key side chains, His164, Phe165 and Asp179, with corresponding bond lengths of 2.460 Å, 2.229 Å and 2.484 Å. At the same time, the S atom of compound L_2_ forms two weak hydrogen bonds with Cys88 and Gly180. Because of the strongly binding between compound L_2_ and the catalytic cores of the DUBs, it would be expected that the catalysis of the DUBs would be inhibited in the presence of compound L_1_. Since there is no crystal structure of POH1 available, we used AMSH (a JAMM domain-containing protein) instead of POH1 to dock L_2_ compound. It was found that L_2_ could also bind with the active sites of AMSH, with CDOCKER Interaction Energy of −35.60 kcal/mol (data not shown).

Next we performed biochemical experiments to verify the computational docking results. The activities of DUBs from either cell lysates or 26S proteasome were detected. As shown in Figure 7b, therapeutic dose of CuPT (0.5 μM) yielded inhibitory effect on cell lysate DUB activity to some extent but not as strongly as N-ethylmaleimide (NEM, 2 mM); however, similar to NEM, CuPT (0.5 μM) could completely inhibit the DUB activities of a purified 26S proteasome (Figure 7c). The cleavage of tetraubiquitin chains (Ub4) mediated by 26S proteasome DUBs was also tested to further confirm this effect. K48-liked Ub chains were cleaved in the presence of 26S proteasome and this was efficiently blocked by CuPT (0.5 and 1 μM) (Figure 7d). DUB labelling test was further performed by using UbVS. UbVS is a potent inhibitor against UCHL5 and USP14. We found that CuPT was able to compete with UbVS's binding with both UCHL5 and USP14 in a dose-dependent manner (Figure 7e). These results suggest that CuPT could target 19S DUBs, UCHL5 and USP14.

To further investigate this possibility, we knockdowned each DUB subunit in selected cancer cell lines and then determine whether that could affect the efficacy of CuPT. GFPu-HEK-293 cells were first transfected with siRNA against POH1, UCHL5 or USP14, and then GFPu protein level was detected. As shown in Figure 7f, POH1 knockdown highly induced accumulation of GFPu proteins (lanes 3 vs. 1), USP14 knockdown decreased GFPu protein level (lanes 5 vs. 1) and UCHL5 knockdown did not dramatically affect GFPu level (lanes 7 vs. 1). Importantly, when the above cells were treated with CuPT, the induced GFPu protein accumulation was inhibited in USP14- and UCHL5-knockdowned cells (lanes 6 and 8 vs. 2) which was not apparent in POH1 knockdowned cells. Next HepG2 cells were transfected with equal amount of siRNAs of the three DUBs for 48 hours and the effect of CuPT on K48-linked ubiquitinated protein accumulation was investigated in the DUB-silenced cells. As shown in Figure 7g, the DUB proteins including POH1, UCHL5 and USP14 were highly downregulated with transfection of each siRNA. We found that CuPT-induced K48-linked ubiquitinated protein accumulation was more inhibited in POH1 knockdowned cells than in USP14 and UCHL5 knockdowned cells (Figure 7h). It was further confirmed that knockdown of POH1 but not UCHL5 and USP14 lead to the disassembly of the 19S proteasome from the 20S proteasome (data not shown), consistent to previous report^24^.

### CuPT inhibits tumor growth and the UPS *in vivo*

**Figure 8:**
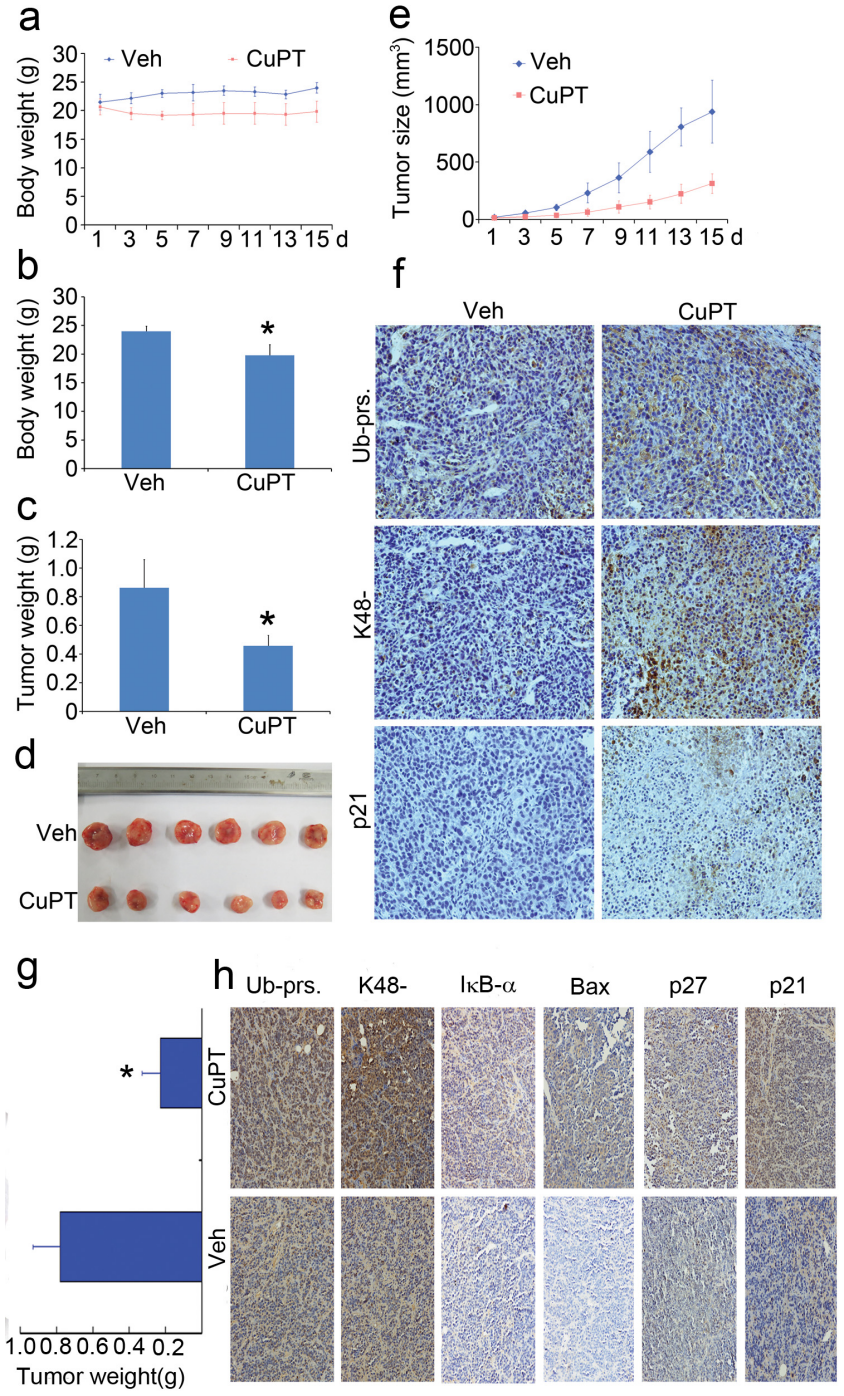
CuPT inhibits tumor growth and the ubiquitin-proteasome system *in vivo*. (a–f) Nude mice bearing HepG2 xenograft tumors were treated with vehicle and CuPT (2.5 mg/kg/d) for totally 15 days (Day 7 interval) after inoculation of HepG2 cells. On day 18 after inoculation, the mice were sacrificed, and the tumor tissues were weighed, imaged and summarized. Body weight was recorded everyday. (a) Body weight changes; (b) Tumor weight and image. *P < 0.05, *versus* CuPT-treated group; (c) Proteasome substrate protein changes. Proteasome-related proteins in tumor tissues were detected by immunohistological analysis. All the immunostaining was repeated in three mouse tumor tissues and the most typical images were shown. (g and h) Nude mice bearing NCI-H929 xenograft tumors were treated with vehicle and CuPT (2.5 mg/kg/d), for 5 days after inoculation of NCI-H929 cells. On day 8 after inoculation, the mice were sacrificed. The tumor tissues were weighed and proteasome-related proteins in tumor tissues were detected by immunohistological analysis.

We further evaluated the effect of CuPT *in vivo* using nude mouse HepG2 and NCI-H929 xenograft models. In HepG2 xenograft model, body weight gradually increased in the control group while kept relatively stable in CuPT-treated group (Figure 8a and b); tumor volume gradually decreased and the tumor weight was significantly reduced in the CuPT treatment group compared to the vehicle control (Figure 8c–e); the immunostaining results showed that the proteasome substrate levels such as total or K48-linked ubiquitinated and p21 proteins were significantly increased in the CuPT-treated tumor tissues (Figure 8f). In NCI-H929 xenograft model, tumor weights were reduced and proteasome-related substrates including total or K48-linked ubiquitinated proteins and p21, p27, Bax, and IκB-α proteins were all significantly increased in the CuPT-treated tumor tissues (Figure 8h). Together, the results showed that CuPT inhibited both tumor growth and the UPS function *in vivo*, consistent to the results *in vitro*.

## Discussion

Recently, several metals, including copper, zinc, gold and gallium among others, have been investigated for their potential as proteasome inhibitors in cancer therapy^8^,^9^,^10^,^11^,^12^,^25^. Thus, metal-based compounds are potential proteasome-inhibitory drug candidates.

It has been found previously that, like other established proteasome inhibitors, copper-binding compounds were only effective in inducing apoptosis in tumor, but not in non-transformed cells, providing great promise in the future clinical trials^6^,^12^. Like dithiocarbamates (DTCs) or clioquinol, PT spontaneously binds with copper salt thus forming an apoptosis and cytotoxic inducer^12^. The combination of PT and CuCl_2_ but not each alone synergistically induced cell death and cytotoxicity with IC_50_ values less than 0.5 μM in multiple cancer cell lines, like in NCI-H929 cancer cells as low as 50 nM. It is known that one of the major reactive products of PT and CuCl_2_ is the formation of 2,2′-dipyridyldisulfide, (PS)_2_, *via* the oxidation of PT^26^; the other major product is the formation of a chelating product, CuPT. Here we found that the two reactive products both contributed to cytotoxicity induced by the combination treatment of PT and CuCl_2_. According to a previous report^27^, the oxidative product of PT, (PS)_2_, is relatively less toxic than the chelating product of PT, CuPT. Furthermore, it was found that both CuPT and 2,2′-dipyridyldisulfide [(PS)_2_] are toxic to marine polychaete Perinereis nuntia, but (PS)_2_ is much less toxic than CuPT, indicating that CuPT is possibaly the major toxic product from the combination of PT and CuCl_2_.

Similar to the established cancer cell lines, CuPT also induced cytotoxicity in primary cancer cells from AML patients with IC_50_ value averaging 57.03 nM, implying its potential applications in clinical conditions. In the current study, bortezomib treatment decreased cell viability with an average of IC_50_ value 20.50 nM in AML cancer cells and 74.23 nM in normal peripheral monocytes, indicating that bortezomib more selectively killed cancer cells. Similar to bortezomib, CuPT has the same tendency to selectively kill AML cancer cells. In nude mouse xenograft model, CuPT selectively inhibited tumor growth with relatively low toxic effect on body weight. These results demonstrated that CuPT contributed to PT/CuCl_2_ combination-induced cytotoxicity and CuPT is a promising metal-containing drug candidate as regard to cytotoxicities, providing a great potential in future clinical cancer therapy.

Similar to other copper chelating agents, the combination of PT and CuCl_2_ forms a proteasome inhibitor. PT and CuCl_2_ combination induced the accumulation of ubiquitinated proteins and GFPu, a surrogate proteasome substrate, but PT and H_2_O_2_ combination did not induce the accumulation of ubiquitinated proteins, indicating that the oxidative product of PT did not induce UPS malfunction. Further studies confirmed that CuPT, similar to the combination of PT and CuCl_2_, not only induced the accumulation of endogenous and exogenous proteasome substrates in cultured cell lines but also accumulated ubiquitinated proteins in cancer cells from AML, which is comparable to bortezomib treatment. The effective doses used in the current study for UPS inhibition are mostly less than 1 μM, implying that PT is a very potent metal chelator as most of the reported copper chelating agents (like Schiff base, clioquinol, disulfiram, NCI-109268, dithiocarbamates and 8-hydroxylquinoline) inhibit the UPS at doses higher than CuPT and PT/CuCl_2_ complex used in the reported conditions^6^,^7^,^9^,^10^,^12^,^13^,^28^,^29^.

We and others have reported that copper could directly inhibit 20S proteasome peptidase activities. In a test tube, PT itself did not have any inhibitory effect on proteasome CT-like activity, consistent to other report^30^; CuCl_2_ could directly inhibit CT-like activity with an IC_50_ value 3–5.3 μM^6^,^12^; the combination of PT/CuCl_2_ or CuPT selectively inhibited CT-like activity but not caspase-like and trypsin-like activities with a similar level of IC_50_ value ~ 6.0 μM (data not shown). These results demonstrated that PT/CuCl_2_ or CuPT only directly inhibit 20S proteasome activities at much higher doses than their cytotoxic doses in the cell. Therefore, we investigated the effects of CuPT or PT/CuCl_2_ combination on 26S proteasome peptidase activities in live cells. As found, CuPT or PT/CuCl_2_ combination strongly inhibit proteasome peptidase activities with IC_50_ value less than 1 μM, like in U266 and NCI-H929 cells as low as less than 0.25 μM, a 24 ~ fold decrease compared with the IC_50_ value in the test tube, consistent to our previous findings^13^. The mechanism by which CuPT increases more CT-like activity inhibition within the cell than in the test tube remains unknown. We proposed that one of the possibilities is due to copper binding to proteasome β5 subunit in the cell as previous reported^6^,^12^ and the second possibility is due to some cell death affecting the peptidase activity. The exact mechanisms needs to be further investigated.

In the current study, one of the most important findings is that 19S deubiquitinases are potential molecular targets of CuPT. UCHL5 and USP14 belong to the cysteine-containing DUBs^14^ and the Cu atom of the CuPT, theoretically, has the potential to react with SH-containing enzymes^31^. Based on the *in silicon* model, CuPT has the potential to interact with both UCHL5 and USP14. This has been confirmed by Ub chain disassembly test, active-site-directed labeling experiment and DUB activity assay with CuPT treatment at therapeutic doses like 0.5 μM. The DUB assay system used in this study is mainly suitable for UCH and USP family DUBs. The enzymatic study confirmed that CuPT at 0.5 μM dose, similar to NEM, could almost completely inhibit the proteasome UCHL5 and USP14 DUB activity. These results demonstrated that CuPT targets proteasome UCHL5 and USP14. Even though in our computational model CuPT could bind with active sites of AMSH, a JAMM domain-containing protein, since there is no crystal structure of human POH1 available, whether CuPT could interact and inhibit human POH1 needs to be investigated in the future.

The additional experiments by silencing proteasome DUBs also supply some evidence to support its inhibition on 19S proteasome. It has been reported that silencing POH1, but not UCHL5 and USP14 lead to the disassembly of the 19S from the 26S proteasome^24^ and this has also been confirmed in our study (data not shown). POH1 knockdown could dramatically inhibit GFPu protein degradation in HEK-293 cells and induce ubiquitinated protein accumulation in HepG2 cancer cells, and this disassembly of 26S proteasome could mostly blocked the effects of CuPT on protein degradation and ubiquitinated protein accumulation, indicating that CuPT-mediated proteasome malfunction did rely on the existence of intact 26S proteasome. Even though the roles of proteasome UCHL5 and USP14 in the cell are far from being understood, most of the studies up to now suggest the editing function of these two DUBs. In this report, it was found that UCHL5 knockdown itself did not dramatically affect GFPu protein degradation but it could partially block the effect of CuPT on GFPu protein degradation, indicating that UCHL5 plays an important role in CuPT-induced protein inhibition. USP14 inhibition both by either drug like IU1 or siRNA silencing has been confirmed to accelerate protein degradation^24^,^32^, which has also been confirmed in this report; USP14 silencing also affected CuPT-induced GFPu protein degradation. However, in HepG2 cancer cells, either UCHL5 or USP14 knockdown did not abrogate CuPT-mediated ubiquitinated protein accumulation. These results demonstrated that UCHL5 and USP14 possibly play different roles in CuPT-mediated DUB inhibition, rather than in ubiquitinated protein accumulation process. Future studies need to be performed to compare the different roles of proteasome DUBs in protein degradation. Besides targeting proteasome DUBs, CuPT could also inhibit some other non-proteasome DUBs in the cytoplasm as well (Figure 7b), which possibly contributes to some non-proteasome-related cytotoxicities.

PT has been widely used in some other fields for many years. ZnPT is well-known for its use in treating dandruff and seborrhoeic dermatitis^33^. ZnPT and CuPT were also introduced on the market as two new alternatives to tributyltin (TBT) for antifouling paint biocides (in 1991 and 1996, respectively) by Arch Chemicals^34^. Recently it has been reported that ZnPT exerted significant anticancer effects *in vivo* and *ex vivo*^35^, and selectively killed quiescent and rapidly dividing cells in a p53-independent manner^36^. Our study has identified one of the major mechanisms of action of this PT/copper complex on inhibiting tumor cell proteasome.

## Methods

### Materials

CuPT was synthesized in our lab or purchased from Sigma-Aldrich Inc. (St. Louis, MO). Other agents are bortezomib (BD Biosciences, San Jose, CA); NEM, Sigma-Aldrich Inc., St. Louis, MO); Proteasome-Glo™ Chymotrypsin-like, Trypsin-like, Caspase-like Cell-Based Assay kits (Promega Bioscience, Madison, WI); Caspase Inhibitor Z-VAD-FMK (BIOMOL International LP, Plymouth Meeting, PA); Suc-Leu-Leu-Val-Tyr-aminomethylcoumarin (Suc-LLVY-AMC), Z-Leu-Leu-Glu-AMC (Z-LLE-AMC), Boc-Leu-Arg-Arg-AMC (Boc-LRR-AMC), 19S, 20S and 26S human Proteasome, HA-Ubiquitin-Vinyl Sulfone (HA-Ub-VS), K48-linked tetra-ubiquitin Ubiquitin-AMC (U550) (BostonBiochem, Cambridge, MA). Control siRNA, POH1 siRNA (h), UCH-L5 siRNA (h), USP14 siRNA (h) (Santa Cruz Biotechnology, Santa Cruz, CA). Antibodies used in this study were purchased from following sources: anti-ubiquitin (P4D1), p27 (F-8), Bax (B-9), IκB-α (FL), anti-GFP (B-2) (Santa Cruz Biotechnology, Santa Cruz, CA); anti-p21 Waf1/Cip1 (DCS60), anti-caspase3 (8G10), anti-caspase8 (1C12), anti-caspase9 (C9), anti-PARP, anti-CHOP (L63F7), anti-K48-linkage specific polyubiquitin (D9D5) (Cell Signaling Technology, Beverly, MA, USA); anti-POH1, anti-UCHL7/UCH37 (Epitomics); anti-USP14 (C-term) (ABGENT); anti-GAPDH, anti-c-Jun (N85), anti-HA-tag (Bioworld Technology, Inc.). MTS assay (CellTiter 96 Aqueous One Solution reagent) was purchased from Promega Corporation (Madison, WI, USA). PI and Annexin V-FITC apoptosis Detection Kit were purchased from Keygen Company (Nanjing, China). Enhanced chemiluminescence (ECL) reagents were purchased from Santa Cruz Biotechnology Inc. (Santa Cruz, CA). Lipofectamine™ RNAiMAX was purchased from Invitrogen Corporation.

### Cell viability assay

MTS assay (CellTiter 96Aqueous One Solution reagent; Promega, Shanghai, China) was used to test cell viability according to previously reported^37^. Briefly, 2 × 10^5^/ml cells in 100 μl were treated with either vehicle or CuPT and other agents for 24 hours. 4 hours before culture termination, 20 μl MTS was added to the wells. The absorbance density was read on a 96-well plate reader at wavelength 490 nm. IC_50_ values were calculated.

### Cell death assay

Apoptosis was determined by flow cytometry using Annexin V-fluoroisothiocyanate (FITC)/PI double staining^37^. Cells were incubated with GA, then collected and washed with binding buffer (Sigma-Aldrich, St. Louis, MO), then incubated in working solution (100 μl binding buffer with 0.3 μl Annexin V-FITC) for 15 min in dark. Cells were washed and resuspended with binding buffer. PI was added just before flow cytometric analysis. Annexin V/PI staining was also performed as described but *in situ*. The double stained cells were also imaged with an inverted fluorescence microscope equipped with a digital camera (Axio Obsever Z1, Zeiss, Germany).

To monitor temporal changes in the incidence of cell death in the live culture condition, PI was added to the cell culture medium, and at the desired sequential time points, the cells in the culture dish were imaged with an inverted fluorescence microscope^38^.

### Western blot analysis

Whole cell lysates were prepared in RIPA buffer (1 × PBS, 1% NP-40, 0.5% sodium deoxycholate, 0.1% SDS) supplemented with 10 mM β-glycerophosphate, 1 mM sodium orthovanadate, 10 mM NaF, 1 mM phenylmethylsulfonyl fluoride (PMSF), and 1 × Roche Complete Mini Protease Inhibitor Cocktail (Roche, Indianapolis, IN). Western blotting was performed as previously described^39^. In brief, an equal amount of total protein extracts from cultured cells were fractionated by 12% SDS-PAGE and electrically transferred onto polyvinylidene difluoride (PVDF) membranes. Primary antibodies and horseradish peroxidase-conjugated appropriate secondary antibodies were used to detect the designated proteins. The bounded secondary antibodies on the PVDF membrane were reacted to the ECL detection reagents (Amersham Bioscience, Dübendorf, Switzerland) and exposed to X-ray films (Kodak, Japan).

### Cell culture and sample collection

Peripheral blood samples of normal controls were obtained from Guangzhou Blood Center and peripheral bone marrow samples of AML patients were obtained from discarded material utilized for routine laboratory tests at the Department of Hematology, Guangzhou First Municipal People's Hospital of Guangzhou Medical University; The use of these materials is approved by the Ethics Committee of these two Institutions with the permission of the patients and volunteers. Totally six patients with AML and six volunteers were recruited in this preclinical study. Mononuclear cells were isolated by Ficoll-Paque (Pharmacia, Uppsala, Sweden) density gradient. Mononuclear cell fraction was cultured in RPMI 1640 culture medium with 15% FBS.

### Peptidase activity assay

About 4,000 cells were treated with GA at various concentrations at 37°C for 6 hours. The drug-treated cells were then incubated with the Glo Cell-Based Assay Reagent (Promega Bioscience, Madison, WI) for 10 minutes. The proteasome peptidase activity was detected as the relative light unit (RLU) generated from the cleaved substrate in the reagent following the manual. Luminescence generated from each reaction was detected with luminescence microplate reader (Varioskan Flash 3001, Thermo, USA). *In vitro* proteolytic peptidase (CT-like peptidase, caspase-like and trypsin-like) activities were detected by using the synthetic fluorogenic peptide substrates as we reported previously^37^.

### Computational modeling

In order to obtain valuable binding information of copper pyrithione toward the DUBs mainly including USP14 and UCHL5, molecular docking studies were performed with CDOCKER protocol of Discovery Studio 2.0 [Accelrys Software Inc. (2007)]. The crystallographic structures of USP14 and UCHL5 were directly downloaded from the Protein Data Bank (PDB IDs: 2AYO and 3RIS). After removing irrelevant components, hydrogen atoms were added and their positions were minimized with a 0.01 kcal/mol/Å root mean square gradient by using the all-atom CHARMm forcefield and the Adopted Basis Newton-Raphson (NR) Algorithm. In addition, taking into account the possible hydrolysis of compound PT (L1) in certain physiological conditions, the hydrolysate PT (L2) was selected as the docking ligand. The geometry structure of compound L2 was optimized using the DFT calculations at the B3LYP/LANL2DZ level to obtain NPA charges by using the Gaussian 03 [Revision D.01,Gaussian, Inc., Wallingford CT (2004)]. During the whole docking process, the two proteins were rigid, while the ligand L2 was flexible. The Input Site Spheres of 12Å radius were centered on each active pocket of USP14 and UCHL5, with (x, y, z) = (38.12, 84.32, 6.61) and (−9.40, 6.57, 61.54), respectively. The conformation corresponding to the lowest CDOCKER Interaction Energy was selected as the most probable binding conformation. All parameters used in calculation were default except for explained.

### Deubiquitinase activity assay

This was performed as reported^40^. Briefly, cell lysate (5 μg) or 26S proteasome (25 nM) was solved in ice-cold DUB buffer containing 50 mmol/L Tris-HCl (pH 7.5), 250 mM sucrose, 5 mM MgCl_2_, and 1 mM PMSF and pretreated with Aur (2 μM) or 2 mM NEM for 15 minutes, then incubated with Ub-AMC substrate in a 100 μL reaction volume at 25°C. AMC release generated from the cleaved substrate was temporally recorded with microplate reader (Varioskan Flash 3001, Thermo, USA).

### Ubiquitin chain disassembly

*In vitro* disassembly of purified polyubiquitin chains (K48-linked) was performed as described earlier^41^. Purified 26S proteasome (25 nM) preincubated with either vehicle or CuPT for 10 min *in vitro*, and then K48-linked chains (1 μg) were added into the reaction DUB buffer for 30 min at 37°C. The extent of chain disassembly was assessed by Western blot.

### DUB labeling assays

26S proteasome (25 nM) was treated with CuPT for 10 minutes and incubated with HA-UbVS for 1 hour at 37°C, followed by boiling in reducing sample buffer and resolving by SDS-PAGE. After protein transfer to PVDF membranes, HA immunoblotting was used to detect DUB labeling^42^.

### SiRNA transfection

Three siRNAs against human POH1, UCHL5 and USP14, constructed and ordered from Guangzhou Ribobio Co. Ltd, were used to transfect HepG2 and HEK-293 cells. For each transfection sample, oligomer Lipofectamine™ complexes were prepared. The oligomer Lipofectamine™ complexes were added to each well containing cells and medium, followed by gentle mixing by rocking the plate back and forth. Medium was changed after 6 hours, and the cells were incubated at 37°C in a CO_2_ incubator for 48 hours, followed by CuPT treatment as indicated. Cells were collected for Western blot assay.

### Nude mouse xenograft model

All animal protocols used were approved by the Institutional Animal Care and Use Committee of Guangzhou Medical University. The mice were obtained from Guangdong Laboratory Animal Monitoring Institute (SCXK2008-2002). The nude Balb/c mice were housed in barrier facilities with a 12 h light dark cycle, with food and water available ad libitum. 3 × 10^7^ of HepG2 cells was inoculated subcutaneously on the flanks of 5-week-old male nude mice. After 72 h of inoculation, mice were treated with either vehicle (10% DMSO, 30% Cremophor ELand 60% NaCl) or CuPT (2.5 mg/kg/day) for totally 15 days (Day 7 interval), respectively. Tumor volumes were recorded and calculated as previously reported^43^.

### Immunohistochemical staining

Formalin-fixed xenografts were embedded in paraffin and sectioned according to standard techniques as we previously reported^43^. Tumor xenograft sections (4 μm) were immunostained using the MaxVision kit (Maixin Biol) according to the manufacturer's instructions. The primary antibodies were used as indicated. 50 μl MaxVisionTM reagent was applied to each slide. Color was developed with 0.05% diaminobenzidine and 0.03% H_2_O_2_ in 50 mM Tris-HCl (pH 7.6), and the slides were counterstained with hematoxylin. A negative control for every antibody was also included for each xenograft specimen by substituting the primary antibody with preimmune rabbit serum.

### Statistical analysis

All the results were expressed as Mean ± SD where applicable. GraphPad Prism 4.0 software (GraphPad Software) was used for statistical analysis. Student's *t* test was used to compare the differences between variables. *P* value of < 0.05 was considered statistically significant.

## Author Contributions

N.L., C.L., X.F.L., H.H., L.G., S.Y., X.Y.L., X.C. and X.H. planned most of the *in vitro* experiments; N.L., W.S. and C.Y. performed the *in vivo* experiments; X.F.L., S.T.L., S.W. and P.Z. mainly performed the *ex vivo* experiments; S.Y.L., C.Z. and P.Z. performed the computational analysis; J.L., S.Y.L., X.W. and Q.P.D. conceived of the study, analyzed data and wrote the manuscript. All authors reviewed the manuscript.
